# Supplementary material for: A novel computational pipeline for var gene expression augments the discovery of changes in the Plasmodium falciparum transcriptome during transition from in vivo to short-term in vitro culture
Source: eLife. 2024 Jan 25;12:RP87726. doi: 10.7554/eLife.87726 (PMC10945709; doi:10.7554/eLife.87726)
Supplement: Supplementary file 2. — Patient exposure and severity refer to the original patient exposure and severity that the sample originated from. # var transcripts before SSPACE represents the # var transcripts assembled before SSPACE was applied. # var transcripts after SSPACE represents the # var transcripts assembled after SSPACE was used to join contigs. # var significant annotated transcripts ≥ 500 nt represent the # var transcripts ≥ 500 nt that contained at least one significantly annotated var domain. # var significant annotated transcripts ≥ 1500 nt and three domains represent the # var transcripts ≥ 1500 nt that contained at least three significantly annotated var domains. Var largest transcript (nt) represents the length of the longest assembled var transcript in that sample in nucleotides. Var N50 represents the length of the shortest var transcript where all transcripts greater than or equal to this length when summed together represent 50% of the total var transcript assembly length. # var transcripts ≥ 5% represents the number of var transcripts whose expression contributed to >5% overall var gene expression. # assembled LARSFADIG represents the number of assembled LARSFADIG motifs (assembled using rnaSPAdes). [file elife-87726-supp2.docx]

**Supplementary file 2. Per sample *var* assembly results using the whole transcript approach.** Patient exposure and severity refer to the original patient exposure and severity that the sample originated from. # *var* transcripts before SSPACE represents the # *var* transcripts assembled before SSPACE was applied. # *var* transcripts after SSPACE represents the # *var* transcripts assembled after SSPACE was used to join contigs. # *var* significant annotated transcripts > =500nt represents the # *var* transcripts >= 500nt that contained at least one significantly annotated *var* domain. # *var* significant annotated transcripts > =1500nt and 3 domains represents the # *var* transcripts > =1500nt that contained at least three significantly annotated *var* domains. *Var* largest transcript (nt) represents the length of the longest assembled *var* transcript in that sample in nucleotides. *Var* N50 represents the length of the shortest *var* transcript where all transcripts greater than or equal to this length when summed together represent 50% of the total *var* transcript assembly length. # *var* transcripts >= 5% represents the number of *var* transcripts whose expression contributed to > 5% overall *var* gene expression. # assembled LARSFADIG represents the number of assembled LARSFADIG motifs (assembled using rnaSPAdes).

| **Sample ID** | **Patient #** | **Sex of patient** | **Generation** | **Patient exposure** | **Patient severity** | **# *var* transcripts before SSPACE** | **# *var* transcripts after SSPACE** | **# Significant *var* transcripts ≥500nt** | **# Significant *var* transcripts ≥1500nt & 3 domains** | ***Var* largest transcript (nt)** | ***Var* N50** | **# *Var* transcripts ≥5%** | **# Assembled LARSFADIG** |
| --- | --- | --- | --- | --- | --- | --- | --- | --- | --- | --- | --- | --- | --- |
| SRR13197346 | 1 | female | *ex vivo* | naive | severe | 161,641 | 161,641 | 16 | 1 | 6,383 | 2,281 | 4 | 2 |
| SRR13197345 | 2 | male | *ex vivo* | pre-exposed | non-severe | 184,373 | 184,373 | 19 | 3 | 5,665 | 5,129 | 2 | 4 |
| SRR13197323 | 4 | male | *ex vivo* | pre-exposed | non-severe | 65,961 | 65,955 | 15 | 2 | 7,726 | 5,908 | 4 | 1 |
| SRR13197320 | 5 | male | *ex vivo* | pre-exposed | non-severe | 47,979 | 47,977 | 61 | 26 | 11,337 | 5,831 | 4 | 14 |
| SRR13197319 | 6 | female | *ex vivo* | naïve | non-severe | 31,669 | 31,661 | 53 | 7 | 7,237 | 1,849 | 4 | 8 |
| SRR13197318 | 7 | male | *ex vivo* | pre-exposed | non-severe | 34,338 | 34,332 | 47 | 17 | 12,287 | 6,069 | 5 | 16 |
| SRR13197317 | 9 | female | *ex vivo* | naïve | non-severe | 68,101 | 68,100 | 83 | 31 | 9,693 | 6,169 | 3 | 20 |
| SRR13197344 | 12 | male | *ex vivo* | pre-exposed | non-severe | 38,035 | 38,026 | 99 | 30 | 7,572 | 5,138 | 4 | 12 |
| SRR13197342 | 14 | male | *ex vivo* | naïve | non-severe | 110,684 | 110,676 | 163 | 68 | 11,975 | 5,637 | 2 | 21 |
| SRR13197339 | 17 | female | *ex vivo* | pre-exposed | non-severe | 115,787 | 115,766 | 48 | 8 | 7,405 | 2,566 | 1 | 13 |
| SRR13197330 | 25 | female | *ex vivo* | naïve | severe | 43,335 | 43,326 | 84 | 46 | 11,475 | 7,011 | 2 | 22 |
| SRR13197329 | 26 | male | *ex vivo* | naïve | severe | 44,777 | 44,767 | 151 | 36 | 10,918 | 5,060 | 2 | 23 |
| SRR13197326 | 29 | male | *ex vivo* | pre-exposed | non-severe | 33,800 | 33,785 | 44 | 20 | 10,440 | 5,836 | 2 | 12 |
| SRR25659814 | 1 | female | 1 | naïve | severe | 19,902 | 19,901 | 61 | 9 | 8,648 | 3,449 | 3 | 9 |
| SRR25659802 | 2 | male | 1 | pre-exposed | non-severe | 32,014 | 32014 | 25 | 5 | 11,607 | 5,768 | 5 | 6 |
| SRR25659796 | 4 | male | 1 | pre-exposed | non-severe | 11,583 | 11,583 | 24 | 10 | 8,462 | 7,024 | 4 | 6 |
| SRR25659794 | 5 | male | 1 | pre-exposed | non-severe | 7,876 | 7,876 | 54 | 15 | 7,833 | 5,605 | 3 | 6 |
| SRR25659793 | 6 | female | 1 | naïve | non-severe | 6,765 | 6,764 | 45 | 20 | 11,345 | 6,408 | 4 | 7 |
| SRR25659812 | 7 | male | 1 | pre-exposed | non-severe | 7,316 | 7,312 | 26 | 10 | 11,369 | 5,987 | 3 | 6 |
| SRR25659810 | 9 | female | 1 | naïve | non-severe | 8,840 | 8,839 | 38 | 2 | 5,989 | 1,067 | 3 | 6 |
| SRR25659808 | 12 | male | 1 | pre-exposed | non-severe | 7,089 | 7,087 | 100 | 29 | 9,816 | 4,348 | 2 | 13 |
| SRR25659806 | 14 | male | 1 | naïve | non-severe | 11,776 | 11,775 | 138 | 38 | 10,219 | 4,622 | 2 | 23 |
| SRR25659804 | 17 | female | 1 | pre-exposed | non-severe | 7,431 | 7,430 | 67 | 25 | 8,996 | 5,301 | 2 | 14 |
| SRR25659801 | 25 | female | 1 | naïve | severe | 13,576 | 13,576 | 85 | 31 | 10,454 | 5,571 | 3 | 12 |
| SRR25659800 | 26 | male | 1 | naïve | severe | 5,833 | 5,833 | 76 | 16 | 8,452 | 4,611 | 2 | 13 |
| SRR25659799 | 29 | male | 1 | pre-exposed | non-severe | 10,424 | 10,423 | 65 | 15 | 7,101 | 4,223 | 3 | 13 |
| SRR25659813 | 1 | female | 2 | naïve | severe | 8,614 | 8,614 | 70 | 15 | 11,067 | 4,024 | 2 | 15 |
| SRR25659797 | 2 | male | 2 | pre-exposed | non-severe | 7,874 | 7,874 | 43 | 6 | 6,088 | 2,282 | 4 | 9 |
| SRR25659795 | 4 | male | 2 | pre-exposed | non-severe | 8,531 | 8,528 | 32 | 7 | 11,306 | 5,648 | 4 | 1 |
| SRR25659792 | 6 | female | 2 | naïve | non-severe | 5,628 | 5,627 | 46 | 11 | 8,547 | 5,486 | 3 | 10 |
| SRR25659811 | 7 | male | 2 | pre-exposed | non-severe | 7,260 | 7,253 | 78 | 36 | 11,412 | 5,567 | 3 | 18 |
| SRR25659809 | 9 | female | 2 | naïve | non-severe | 13,341 | 13,340 | 73 | 16 | 7,477 | 3,166 | 2 | 10 |
| SRR25659807 | 12 | male | 2 | pre-exposed | non-severe | 10,026 | 10,024 | 153 | 33 | 6,775 | 2,774 | 3 | 10 |
| SRR25659805 | 14 | male | 2 | naïve | non-severe | 9,615 | 9,612 | 159 | 54 | 9,079 | 4,472 | 3 | 27 |
| SRR25659803 | 17 | female | 2 | pre-exposed | non-severe | 9,303 | 9,301 | 55 | 12 | 8,628 | 4,087 | 4 | 7 |
| SRR25659798 | 29 | male | 2 | pre-exposed | non-severe | 16,306 | 16,304 | 80 | 20 | 7,164 | 3,468 | 4 | 7 |
| SRR25659791 | 6 | female | 3 | naïve | non-severe | 7,577 | 7,573 | 75 | 18 | 7,895 | 3,418 | 3 | 10 |
