# Supplementary material for: A novel computational pipeline for var gene expression augments the discovery of changes in the Plasmodium falciparum transcriptome during transition from in vivo to short-term in vitro culture
Source: eLife. 2024 Jan 25;12:RP87726. doi: 10.7554/eLife.87726 (PMC10945709; doi:10.7554/eLife.87726)
Supplement: Supplementary file 3. — The var transcripts were assembled using the whole transcript approach and all samples’ assemblies combined into a reference. The first approach filtered for var transcripts that contained at least three significantly annotated domains, one of which had to be DBLα and required the transcript to be ≥ 1500 nt in length (3 domains, ≥ 1500 nt and DBLα). The second approach filtered for var transcripts at least 1500 nt long and that contained a DBLα domain (≥1500 nt and DBLα). The third approach filtered for transcripts that contained at least three significantly annotated var domains and were at least 1500 nt in length (3 domains & ≥ 1500 nt). # Significantly annotated var transcripts represent the number of significantly annotated var transcripts in all samples combined. # Uniquely annotated var transcripts represent the number of unique var transcript annotations found in all samples combined. # Var transcripts (≥5 in at least 3 samples) represent the number of var transcripts after filtering for a Salmon estimated count of 5 in at least 3 samples (filtering threshold used prior to differential expression analysis). Max length of var transcript (nt) represents the longest transcript assembled in all samples combined. N50 represents the length of the shortest var transcript where all transcripts greater than or equal to this length when summed together represent 50% of the total var transcript assembly length. Transcripts were annotated using hidden Markov models (HMM) built on the Rask et al., 2010, dataset. When annotating the whole transcript, the most significant alignment was taken as the best annotation for each region of the assembled transcript (e-value cut-off 1e-5). Multiple annotations were allowed on the transcript if they were not overlapping, determined using cath-resolve-hits. [file elife-87726-supp3.docx]

**Supplementary file 3. Summary statistics of the *var* transcripts after 3 different filtering approaches were applied to the paired *ex vivo* (n =13), generation 1 (n=13), generation 2 (n=10) and generation 3 (n=1) samples.** The *var* transcripts were assembled using the whole transcript approach and all samples’ assemblies combined into a reference. The first approach filtered for *var* transcripts that contained at least 3 significantly annotated domains, one of which had to be DBLα and required the transcript to be >= 1500nt in length (3 domains, >= 1500 nt & DBLα). The second approach filtered for *var* transcripts at least 1500nt long and that contained a DBLα domain (>= 1500 nt & DBLα). The third approach filtered for transcripts that contained at least 3 significantly annotated *var* domains and were at least 1500nt in length (3 domains & >= 1500 nt). # Significantly annotated *var* transcripts represents the number of significantly annotated *var* transcripts in all samples combined. # Uniquely annotated *var* transcripts represents the number of unique *var* transcript annotations found in all samples combined. # *Var* transcripts (> =5 in at least 3 samples) represents the number of *var* transcripts after filtering for a Salmon estimated count of 5 in at least 3 samples (filtering threshold used prior to differential expression analysis). Max length of *var* transcript (nt) represents the longest transcript assembled in all samples combined. N50 represents the length of the shortest *var* transcript where all transcripts greater than or equal to this length when summed together represent 50% of the total *var* transcript assembly length. Transcripts were annotated using HMM models built on the Rask et al., 2010 dataset. When annotating the whole transcript, the most significant alignment was taken as the best annotation for each region of the assembled transcript (e-value cut off 1e-5). Multiple annotations were allowed on the transcript if they were not overlapping, determined using cath-resolve-hits.

|  | **3 domains, ≥1500 nt & DBLα** | **≥1500 nt & DBLα** | **3 domains, ≥1500 nt** |
| --- | --- | --- | --- |
| **# Significantly annotated *var* transcripts** | 543 | 568 | 746 |
| **# Unique significantly annotated *var* transcripts** | 313 | 330 | 459 |
| **# *Var* transcripts (≥5 counts in at least 3 samples)** | 309 | 161 | 455 |
| **Maximum length of *var* transcripts (nt)** | 12,287 | 12,287 | 12,287 |
| **N50** | 6,005 | 5,983 | 5,858 |
