# Supplementary figures and images for: A novel computational pipeline for var gene expression augments the discovery of changes in the Plasmodium falciparum transcriptome during transition from in vivo to short-term in vitro culture
Source: eLife. 2024 Jan 25;12:RP87726. doi: 10.7554/eLife.87726 (PMC10945709; doi:10.7554/eLife.87726)

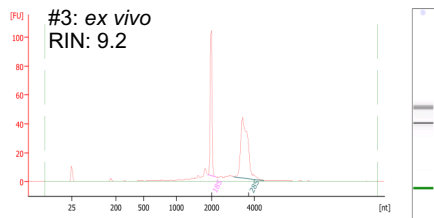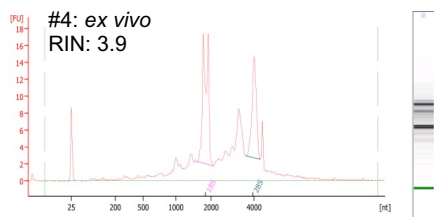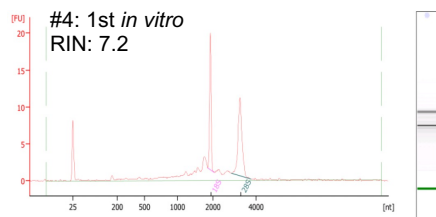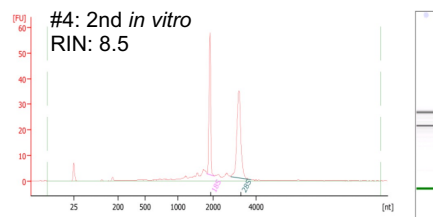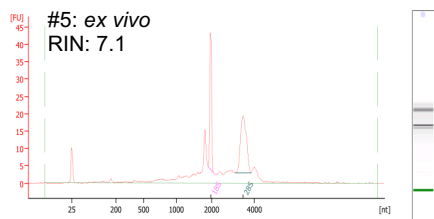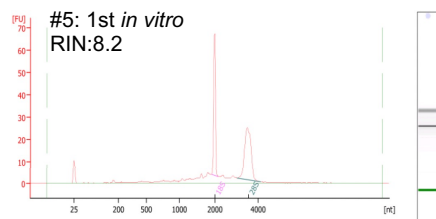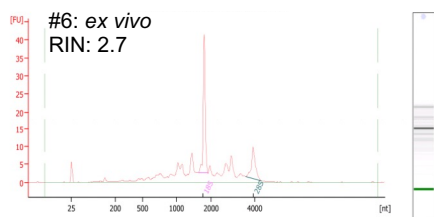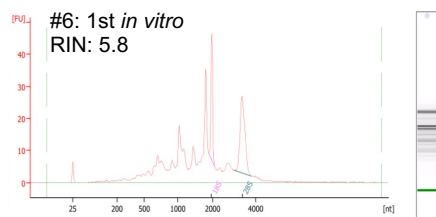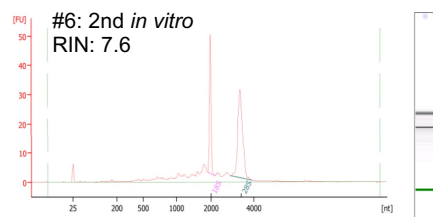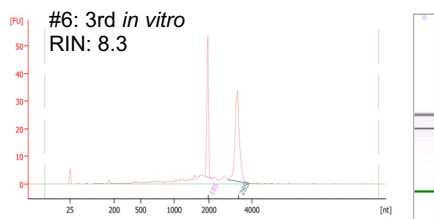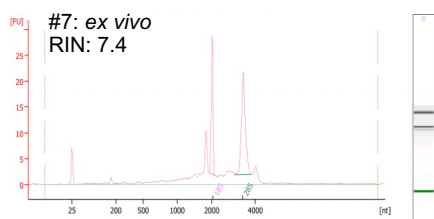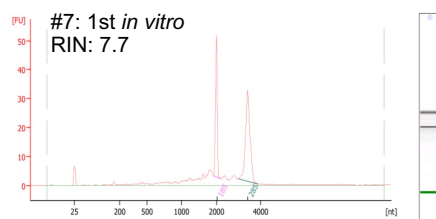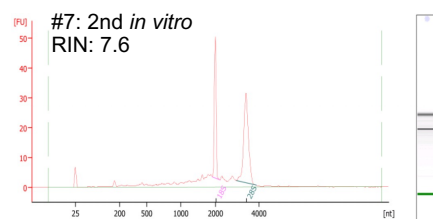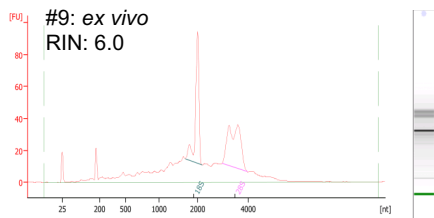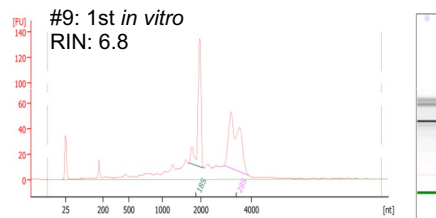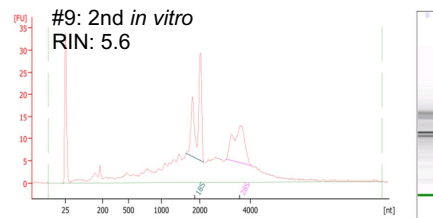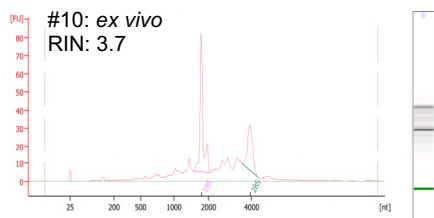

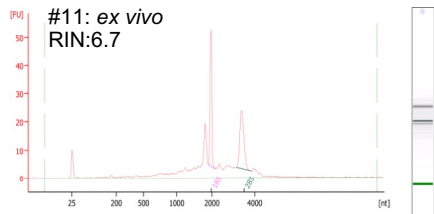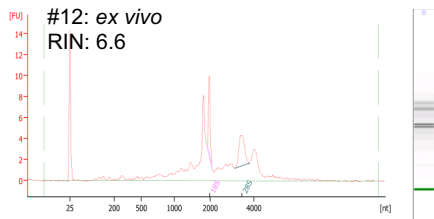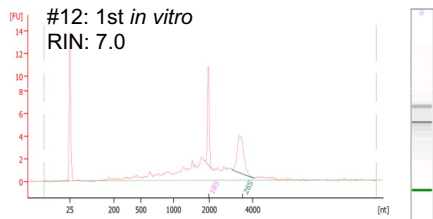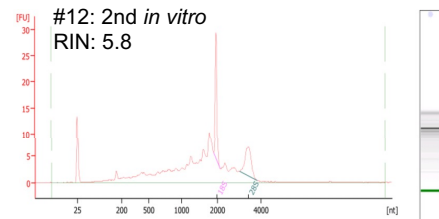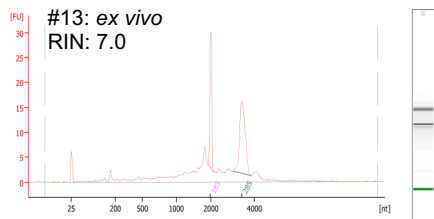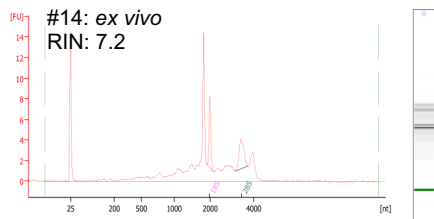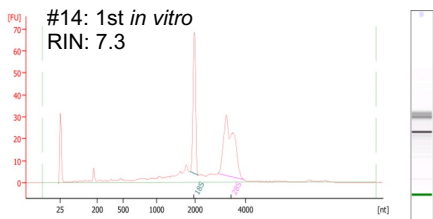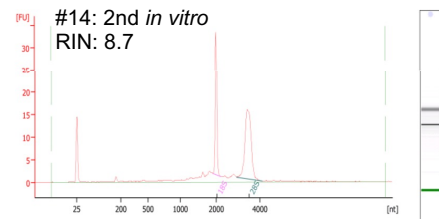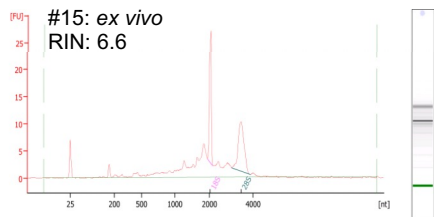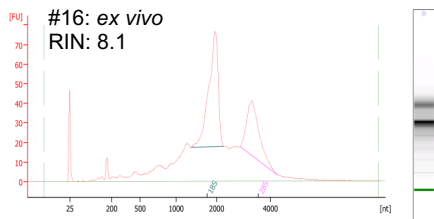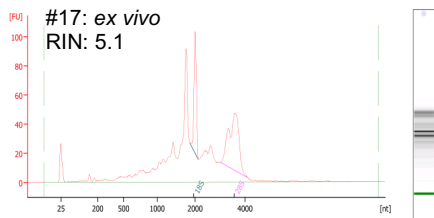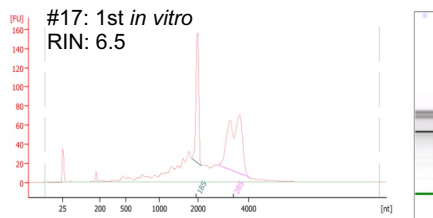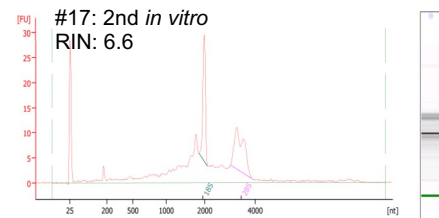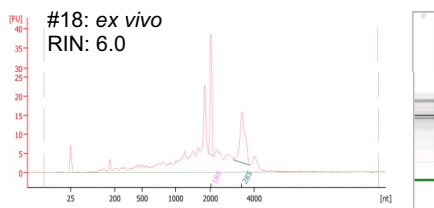

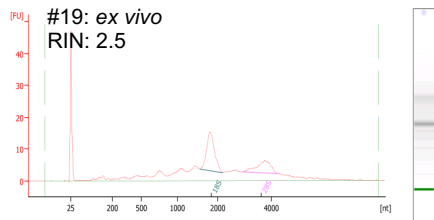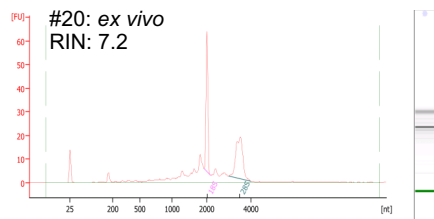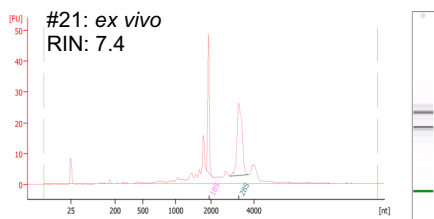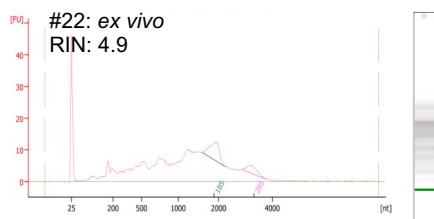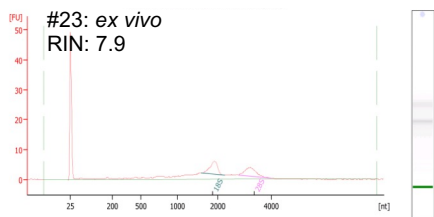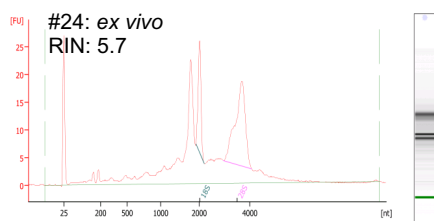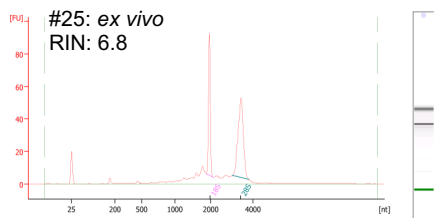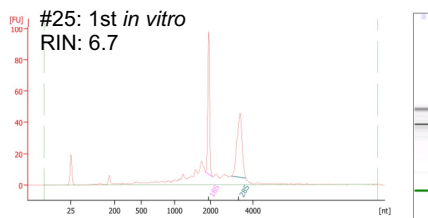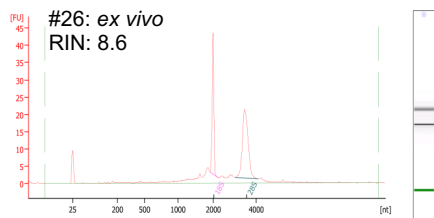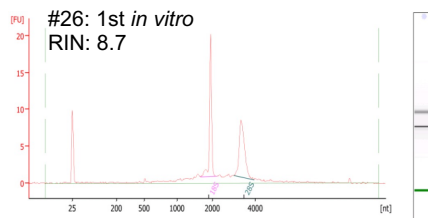

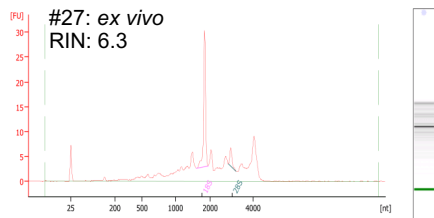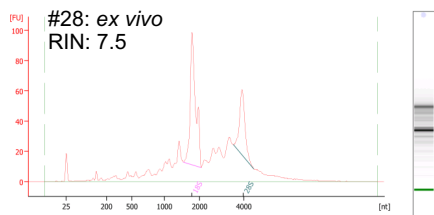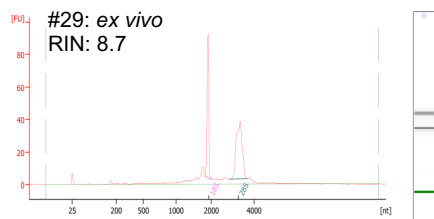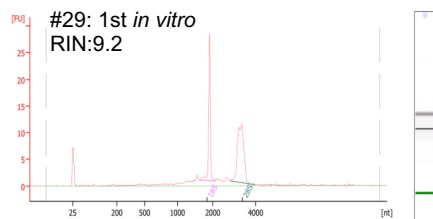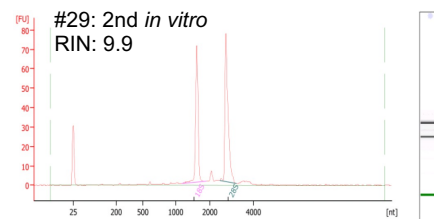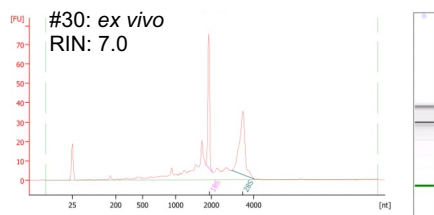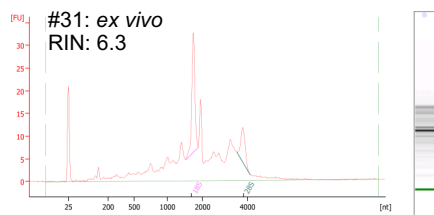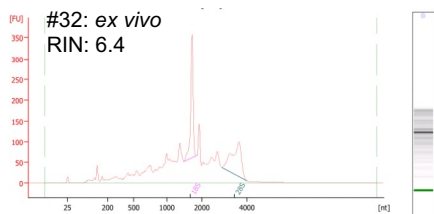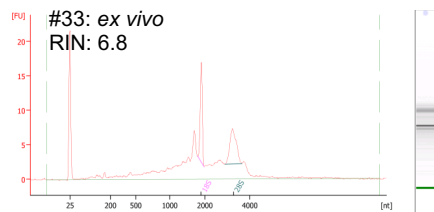

Supplement: Supplementary file 9. — To characterise the overall RNA quality prior to library synthesis, the Bioanalyzer automated RNA electrophoresis system was used to visualise the samples and calculate the RIN values. The measurement of the RIN value for samples from mixed species (Homo sapiens, P. falciparum) is not very meaningful, as the RIN increases the higher the proportion of a single species. This can be observed by the increase in the RIN value during in vitro cultivation of the parasites, as the parasite RNA content increases over time. Of the four rRNA peaks visible in particular in the ex vivo samples, the inner peaks represent the 18S and 28S rRNA of P. falciparum, the outer peaks are of human origin. [file elife-87726-supp9.pdf]
